# Medical Faculty of Minorca

**Published:** 1843-10

**Authors:** 


*Medical Faculty of Minorca.*—The Faculty at the Balearic Islands are under the direction of the Royal Medical Junto at Madrid, without whose license no one is permitted to practise medicine or surgery within the dominions of Her Catholic Majesty; and all persons violating this law are subject to a heavy penalty which may be imposed by any of the courts. This statute, intended for the promotion of medical science, is in reality, the great obstacle in the path of the advancement of the character of the profession in Spain; for the license can always be purchased in the present crippled state of the public treasury, and the course of professional education and examinations, necessary elsewhere, is not required to procure this royal permission. The medical sub-delegate for Mahon is Dr. F. Hernandez, a gentleman of liberal education and handsome professional acquirements; and there are several other men here, at the head of whom stands Dr. Sancho, whose abilities and gentlemanly deportment would secure to them a high rank, wherever such qualities are appreciated; but amid the ignorance and prejudices of Mahon, their light is under a bushel. These men are eclipsed in their professional celebrity by a host of uneducated and illiterate *Medicos*, who by means of bribes have obtained the royal licence; and, dealing in the nostrums and adopting the popular prejudices of the superstitious natives, they not only monopolize the practice, but what little money is paid for medical advice, is sure to fall into their hands. At the head of these is a man of much natural tact and cleverness, and who has not only placed himself at the head of the practitioners, but has also made himself one of the leading citizens of the Island. In early life he followed the avocation of a barber, which probably initiated him into the gossip and tattle which, even in more refined and fashionable cities, form so necessary a qualification for the success of the physician. To this, he unites great moral courage; he boldly plunges into every operation, and usually succeeds in making it redound to his credit, however fatal it may have been to his patient. It is scarcely necessary to add, that this is the individual who boldly plunged the knife into the popliteal aneurism of Mr. V., before mentioned, which occasioned his premature death. The British consul exhibited to me a urinary calculus, which this surgeon had extracted, a short time before our arrival; but unfortunately the patient, a shoemaker, had been taken from his work-bench, without any preparatory treatment, to the operating table, from which he was carried a lifeless corpse; and the stone was not removed until after his death. This individual monopolizes the most lucrative practice of the city; and with an ostentatious display of his property, throws into the background all his professional opponents. There are but few horses at Mahon, and to be able to keep one is the most conclusive evidence of being a *cavallerio.* This man accordingly makes his daily rounds to his patients, mounted. As Dean Swift says—

> It is indeed a very sorry hack,
> But that's of course,
> For what's expected from a horse,
> With an apothecary on his back.

Carrying heavy holsters and pistols, with a golden band around his cap, this disciple of Æsculapius looks but little like an humble practitioner of the healing art.

In consequence of our introduction to surgical practice in the case of aneurism before mentioned, we were asked to do several operations, which gave us a reputation that would not have followed elsewhere. Cases flowed in upon us with great rapidity, from all directions, and in this way most of the remarkable cases came under our observation. Thus we learned much of the state of the profession, from the treatment pursued in those cases; and among these processes of cure, charms, amulets, and the superstitious influence of the Church, had a large share. The priest sells amulets to ward off and cure the tertian fevers; keeping a cross, they say, made from the fig-tree, over the heart, will have the same effect; and eating salt, while looking at the new moon, will also prevent fevers. We will not deny, however, in view of the influence of mind upon the physical frame, that the recurrence of a tertian, kept up perhaps by habit, may thus be sometimes prevented; but if these practices are not regarded by the reader as absurd in the highest degree, the two following examples will certainly rank as such.

We were called in consultation in the case of the wife of the Dutch consul,—a most estimable lady, with an interesting family,—who, at the time of our seeing her, was almost in *articulo mortis* from intussusception. In this condition, a sheep was brought into her chamber, slaughtered at the side of her bed, the omentum taken warm and reeking from it, and placed upon her abdomen,—a remedy that the native physicians assured the family would give immediate relief. On another occasion we were called to see the child of Orfila, a connexion of the distinguished President of the Medical Faculty of Paris, who is a native of Mahon. We found the little patient in the last stage of hydrocephalus; the pupils were dilated, and sensibility almost destroyed. On the crown of the head, a pigeon had been tied, the poor bird having been cut open and applied warm with the feathers on, the blood at the same time streaming down the little patient's face, imparting the most forbidding aspect, as it lay upon its mother's arms. The doctor, during the same time, was lecturing, in a pompus and boisterous manner, upon the virtues of a warm dove—the emblem of innocence—applied to the head of an innocent babe, to draw out all the bad humors which may have collected there.

The medicines in universal repute for almost every disease which "flesh is heir to," are Morrison's pills and the purgative mixture of Le Roy. The former is for sale at every shop in the country, accompanied with a pamphlet in the Spanish language, printed in London, setting forth, in glowing colors, the incredible virtues of this panacea; all of which is readily believed by the ignorant, who swallow these pills without limit. Many instances of their injurious, nay fatal consequences, came under our observation. Among these was the case of a paymaster in the Spanish army, who, after six years of active service in the field, during the civil war, reached Mahon, worn down and exhausted by the incessant labors and exposure of the camp. Immediately after his arrival, he was placed on the use of Morrison's pills, taking large doses of them daily. The excessive purging increased the prostration under which he had previously labored, and mortification took place in one foot, which, after great sufferings, came off at the ankle. In another case, of an old friar who had suffered for years from asthma, these pills were directed in immense quantities, which were increased as he grew worse. At length, unable to take the pills, they were dissolved and administered in solution; and we were informed by Sintès, a most respectable druggist, that within six days he had taken more than five hundred pills,—a cause quite sufficient to put a period to his existence. Whatever good these pills may have accomplished in England, the evil that they have occasioned in this little Island will more than counterbalance; and it is much to be regretted, that such remedies are sent abroad, with all the emblazonry of noble patronage at home, only to deal misery and death among the unfortunate and too credulous, who suppose that, because they come from Britain, where the medical profession are known to possess so much ability, they must necessarily be useful for all the diseases for which they are recommended. Upon the mercenary authors of this wholesale murder rests a fearful and a dreadful responsibility, and although they may escape human laws, yet a retributive justice awaits them hereafter.

Our practice being in every case gratuitous, it soon became extensive; and we rejoiced to have it in our power to assist, by medical advice, the large number who were suffering from the want of it. But when fully engaged in the midst of our career, we were officially notified by most formidable bulletins from the medical sub-delegate, that, in consequence of our not having a licence from the Government at Madrid, we must for the future desist in our practice. At the same time, we were informed by him, that it was, on his part, only a matter of form, which he was compelled to adopt in virtue of his office, and that we might continue to render our services as heretofore. He assured us that there would be no further proceedings in the case; and, accordingly, we continued to prescribe and operate whenever necessary; and in every operation of importance, a majority of the faculty, with the medical students of the town, were always present. In a short time, however, we were duly ordered to appear before the chief-justice of the Island, and answer to a long series of charges for operating and rendering medical advice, in various cases duly mentioned, after having been notified by the proper officer to desist.

Trial by jury is here unknown, but fortunately the judge, before whom the case was brought, was our friend in return for some small service rendered to a member of his family. These proceedings at court were conducted with an ingenuity which would have been creditable to a Philadelphia lawyer, or some of the gentlemen of the long robe at Westminster Hall. We will, therefore, mention one or two of the most important points. The first case mentioned in the indictment was for having extracted a cataruct from the eye of Mr. Escudera, in the presence of some ten or twelve of the citizens of Mahon; and although all these gentlemen were present, yet the fact of the operation was not proved to the satisfaction of the court. One did not look on while operating, another did not see the lens extracted, a third did not know the day of the month on which it was done, and a fourth stated that it was in the afternoon and not in the morning, as set forth in the indictment; all of which was deemed sufficient to acquit us of the charge. During the investigation, the judge received several abusive threatening anonymous letters, the authorship of which was attributed to one of the native physicians, who, upon being charged with it, plead "not guilty." The writing was disguised, but, unluckily for the author, the very fact that he did not find an education necessary to the art of physic, served for his conviction; for, as the letter contained many errors of orthography, the judge directed him to write a copy of the letter as it was dictated to him in court; and, when compared with the anonymous letter, unfortunately for him, every error was found to correspond precisely, notwithstanding the handwriting was entirely different.

The decision of the court was in our favor, and the cost, amounting to forty dollars, was ordered to be paid by the Medical Association of the place; but this event was followed by an open declaration of war on the part of the united faculty, who drove us from the field by the following formidable document, through the hands of O. Rich, Esq., U. S. Consul, and Commodore Isaac Hull:—

"*Subdelegation of the Medical Junto at Mahon.*

"Sir,—It has been repeatedly complained of to the Medical Subdelegate of Mahon, that you gave and still give advice, and practice as a Physician and Surgeon among the citizens of Her Catholic Majesty's dominions, without the Royal licence. You, are, therefore, hereby notified, positively and peremptorily, to discontinue all such practice for the future; and you are not permitted to perform the functions of a Physician or Surgeon to any other individuals than the Anglo-American citizens of your own country.

"I kiss your hand,

"F. HERNANDEZ, *Medical Subdelegate.*

"J. M. Foltz, *Surgeon U. S. Navy.*

After the reception of this missile, we declined giving our advice to such as applied for it; but a few days after, a child was brought to us from Barcelona, in Spain, with club feet, which was of proper age and in an excellent condition for an operation. At the same time, a lieutenant of the Spanish army applied for aid, with a permission from the governor for us to prescribe in his case, which we offered cheerfully to do, on condition that permission would be also given to operate on the child; but this his excellency refused to grant. Still desirous to relieve the child, which had undergone the dangers of a voyage at sea for the purpose of having this operation done; we proposed to operate on board one of the ships of war in the harbor; but the father, a British subject and a man of some property, prudently declined, as he was convinced should we operate after the positive refusal of the governor, that the opportunity would be gladly seized upon to impose on him a heavy fine. The child was, therefore taken to Marseilles, at the hazard of another voyage at sea, to have an operation done, which could have been performed here in two or three minutes.

We have been particular in detailing these cases, as they will not only give a correct idea of the condition of the medical profession in Spain, but will also apprise the reader of the oppression of the government. The only hospital or charitable institution at Mahon is an hospital for foundlings, which is liberally endowed, having at this time about one hundred names upon its register entitled to its charities, among whom are twenty-seven children. Every child deposited here can ever after claim the shelter of the institution; and, although usually placed out to masters as soon as they reach a proper age, yet they ever look to this establishment as their home in after life. There is throughout the Island no dispensary for the distribution of medicines to the poor; and, so far as we could judge, there is but little charity to them from the medical men. They are, indeed, compelled by their own wants to ask some remuneration for their services, insisting usually upon some fee, and being willing to take the most trifling amount rather than go away empty-handed. As we were ordered, shortly after these doctors' quarrels, for service afloat, the little ill-will occasioned for the moment was soon "in the deep bosom of the ocean buried." We shall, indeed, ever rejoice to hear of their individual and united prosperity, for there are among them many of whom we shall ever have the most agreeable and pleasing recollections, and we shall pray that the causes which have occasioned their present humble position may speedily be removed.—*Dr. Foltz* in *New York Journ. of Medicine.*

